# Effect of Vitamin E on Transcriptomic Alterations in Alzheimer’s Disease

**DOI:** 10.3390/ijms241512372

**Published:** 2023-08-03

**Authors:** Sophia Ogechi Ekeuku, Nuraqila Mohd Murshid, Siti Nursyazwani Shukri, Nur Fatin Nabilah Mohd Sahardi, Suzana Makpol

**Affiliations:** Department of Biochemistry, Faculty of Medicine, Universiti Kebangsaan Malaysia, Level 17, Preclinical Building, Jalan Yaacob Latif, Bandar Tun Razak, Cheras, Kuala Lumpur 56000, Malaysia

**Keywords:** vitamin E, transcriptomics changes, Alzheimer’s disease

## Abstract

Research into ageing is focused on understanding why some people can maintain cognitive ability and others lose autonomy, affecting their quality of life. Studies have revealed that age-related neurodegenerative disorders like Alzheimer’s disease (AD) are now major causes of death among the elderly, surpassing malignancy. This review examines the effects of vitamin E on transcriptomic changes in ageing and neurodegenerative diseases, using AD as an example, and how different transcriptome profiling techniques can shape the results. Despite mixed results from transcriptomic studies on AD patients’ brains, we think advanced technologies could offer a more detailed and accurate tool for such analysis. Research has also demonstrated the role of antioxidant modifiers in preventing AD. This review will explore the key findings regarding AD and its modulation by vitamin E, emphasizing the shift in its epidemiology during the ageing process.

## 1. Introduction

The most convincing model of ageing that has been was presented is by Rowe and Kahn [1]. Three things are the cornerstone of their philosophy: a fierce commitment to living, exceptionally high levels of physical and mental functioning, and a low probability of illness and the resulting disability. In contrast to “natural ageing,” productive ageing investigates potential outcomes that might be expected when aiming for promoting better lives [2]. According to observations, human self-renewing tissues divide at a significantly slower rate as people age [3]. In contrast, a previous study found that the rate of cell division in the tissues of mice, regarding the incidence of cancer, did not decrease with age. This discovery has significant implications for how we age, given that little is known about this universal process. The report offers an additional explanation that elucidates the puzzling decline in cancer in the oldest (and fastest-growing) group of Western populations, backed by empirical data [3]. There are four primary ageing features: genome instability, telomeric abrasion, epigenetic alteration and protein homeostasis disruption; this has three antagonistic characteristics, namely unregulated nutrient sensing, mitochondrial dysfunction, and cellular senescence, as well as two integrative traits, namely stem cell depletion and intercellular interaction, all of which result from several fundamental molecular alterations [4]. However, a recent study discovered that several age-related traits, including genomic imbalance, telomere weakening, epigenetic alterations, proteostasis, mitochondrial disruption, cellular senescence, the deregulation of nutrients, stem cell depletion, and altered intercellular communication, are linked to an increased risk of neurodegenerative diseases [5]. As a risk factor for the etiology of neurodegenerative disorders, mitochondrial dysfunction was also identified [6].

Ex vivo and in vivo inferences relate mitochondrial DNA (mtDNA) mutations to the sequence of events in the mitochondrial free radical theory of ageing (MFRTA) and highlight the crucial role of mtDNA mutations in stem cells as the critical event in ageing. The mtDNA mutator mouse, a homozygous mouse produced through a transformation that led to the emergence of the mtDNA polymerase catalytic subunit, is the source of the first research evidence. All of this points to significant mtDNA mutagenesis and several phenotypes associated with natural ageing. In this murine model, many mtDNA point mutations are thought to be the root cause of the instability of the electron transport chain subunits. Both the quantity and quality of stem cells are impacted by high mtDNA mutagenesis, making it more difficult for them to stay in the quiescent state needed for somatic stem cell recovery and long-term survival. In mtDNA mutator mice, the sudden, unexpected onset of the ageing phenotype may be responsible for the early onset of somatic stem cell dysfunction [7]. Based on the notion of free-radical-induced ageing, efforts to prolong life have been performed with two major objectives in mind: modifying the oxidant load or switching up antioxidant mitochondrial capacity. Although mitochondrial antioxidant alternatives are also investigated by inducing genetic mutation or nutritional supplementation, oxidant load adjustments are frequently investigated through calorie reductions [8].

Alzheimer’s disease (AD) is the most common degenerative central nervous system disease in the elderly and the leading cause of dementia [9]. It is characterized by the progressive loss of synapses and neurons in the hippocampus and cortex, resulting in a gradual decline in short-term memory and cognitive functions [10]. According to the Alzheimer’s Association, Alzheimer’s disease accounts for 60–80% of dementia cases [11]. With each year that passes, the frequency of AD has increased significantly, as it now affects over 36 million people worldwide; the number of people affected by Alzheimer’s disease is expected to triple by 2050 [12,13]. An important factor in ageing and degenerative illnesses is cellular senescence, a biological homeostatic system. Senescent cells have been found to accumulate in the brain system with ageing and neurodegenerative illnesses, which may predispose a person to acquiring or worsening a neurodegenerative condition [14].

β-amyloid in senile plaques is thought to be the initiating factor in AD pathology. β-amyloid deposited in the hippocampus and basal segment as neurotoxic amyloid plaques attracts more β-amyloid to form insoluble aggregates, causing mitochondrial damage, unstable homeostasis, and synaptic dysfunction. Microglia and astrocytes are activated, causing inflammatory and oxidative reactions. Neuronal dysfunction and apoptosis eventually occur, leading to AD [9]. In the astrocytes and microglia of the AD brain, it was discovered that β-amyloid plaques increased reactive astrocytes. As a result, amyloid precursor protein, β-secretase (BACE1), and γ-secretase, the three components required for Aβ production, will increase [15]. Currently, the goals of β-amyloid hypothesis-based therapeutic strategies are to reduce β-amyloid formation and aggregation, while increasing its clearance. Controlling BACE1 and γ-secretase activity is the most direct way to reduce β-amyloid production [9]. However, γ-secretase inhibitors lack APP substrate specificity and are toxic to a variety of organs [9]. Recently, researchers at the University of Washington created small synthetic alpha-peptides that target and inhibit small toxic oligomers while also preventing Aβ aggregation from occurring. In AD mouse disease models and Caenorhabditis elegans (an AD nematode model), good results were obtained. However, it remains to be seen whether this technique can be used on humans [16].

Another complex multifactorial process involved in AD pathology is tau development. In the brains of Alzheimer’s disease patients, hyperphosphorylated tau causes configuration changes and a loss of tubulin polymerization capacity [9], resulting in impaired microtubule function [17]. Tau–tau interactions and polymerization result in the formation of insoluble paired helical filaments and straight filaments, which result in the formation of intraneuronal fibrillar deposits known as neurofibrillary tangles (NFTs) [9], which reduce synaptic density, cause neurotoxicity and cause cell dysfunction [9,18]. Experiments have shown that tau hyperphosphorylation correlates with the degree of tau aggregation and the pathological severity of Alzheimer’s disease because tau, not β-amyloid, determines cognitive status [9,19]. Recently, kinase and tau aggregation inhibitors, microtubule stabilizers, and immunotherapeutic drugs have been investigated; however, most of them are toxic and ineffective [9,20,21]. AADvac1 as a tau vaccine demonstrated promising results in terms of its safety and immune response in Alzheimer’s disease patients. However, more research is required to prove its clinical efficacy [22].

Antioxidant drug therapy has been investigated as a potential AD treatment due to the significant presence of oxidative damage associated with abnormal β-amyloid accumulation and NFT deposition in the brains of AD patients. Because of their diverse array of physiological actions, including potent antioxidant effects, naturally occurring compounds, such as plant polyphenols, have been suggested to have potential neuroprotective effects against AD [23]. This review will concentrate on the role of oxidative stress in AD, transcriptomic changes associated with AD and how vitamin E affects them. Additionally, it will also discuss how various transcriptomic analysis methods, including RNA-sequencing and microarrays, affect the results.

## 2. Role of Oxidative Stress in Alzheimer’s Disease

Reactive oxygen species (ROS) buildup is a result of damaged mitochondria producing less ATP as people age. According to recent research, ROS may cause oxidative stress (OS) that damages mitochondria and contributes to the bioenergetic crisis that results in neurodegenerative diseases and speeds up ageing [24]. Numerous neurological conditions, including ischemic stroke, Alzheimer’s disease, Parkinson’s disease, mild cognitive impairment (MCI), and amyotrophic lateral sclerosis (ALS), are all linked to oxidative stress [25]. When ROS production exceeds antioxidant defense, an imbalance between free radical production and antioxidant capacity results, causing oxidative stress, which is harmful and impairs cellular function in general. This condition can be found in many pathologies associated with ageing and mitochondrial dysfunction [26].

Being the most active organ in the body, the brain is more susceptible to oxidative stress. Due to its increased oxygen needs, the brain utilizes 20% more oxygen than other bodily components. In the brain, ROS are formed as a result of the concentration of redox-active metals (copper and iron). However, compared to other organs, the brain possesses fewer antioxidant enzymes. As a result, lipid peroxidation is more likely to occur in the PUFA-rich brain cell membranes [27]. Lipid peroxidation may also play a role in the loss of long-term potentiation and other synaptic functions associated with learning and memory in Alzheimer’s disease brains [28]. Furthermore, AD brains were found to have increased neuronal Cu^2+^ and Zn^2+^ levels that were up to three times the physiological value of a healthy control brain. These cations can bind to the hydrophilic N-termini of Aβ peptides, undergo a redox reaction, and generate significant amounts of ROS. As a result, it appears that there is a positive feedback loop of increased oxidation and increased ROS production [23]. Furthermore, copper is pathologically transferred to senile plaques in Alzheimer’s patients, resulting in a deficiency of this metal in brain cells. A further indication of this disturbed metal homeostasis is the dysregulation of physiological functions controlled by metalloenzymes [29]. All these markers of OS have been described as an early alteration in the AD brain, and this concept has previously been used to support the ‘oxidative stress hypothesis’ in the pathogenesis of AD [30].

Due to its connection to the buildup and deposition of β-amyloid, OS induced by ROS is also regarded as one of the key factors in the pathogenesis of AD. It has been observed that OS can be exacerbated in patients with early-stage disease due to the accumulation of β-amyloid, which further causes mitochondrial dysfunction [31]. Studies have revealed that, in contrast to the low levels of antioxidants and their enzymes (such as vitamin C) found in Alzheimer’s patients, the imbalance in OS caused by β-amyloid determines a high level of by-products (such as protein and DNA/RNA oxidation). Additionally, it has been noted that flaws in antioxidants’ defense mechanisms cause high levels of OS, which promote the production and deposition of β-amyloid [31].

A reduction in ATP synthesis is brought on by oxidative modification in AD. It is exhibited in the neurons’ capacity to sustain biological procedures such as synaptic transmission, action potential genesis, and synaptic assembly [28]. There is mounting evidence that mitochondrial dysfunction and oxidative stress are key factors in the emergence of AD [32]. It has also been demonstrated that mtDNA, lipids, and proteins are damaged by elevated oxidative stress in AD brains [33]. Lipid peroxidation is one of the AD processes that can result in the phospholipid disbalance seen in AD patients’ brains. The brains of AD patients typically contain high levels of malondialdehyde (MDA) and four-hydroxynonenal (4-HNE), two other aldehydes. Toxic to neurons, 4-HNE alters the microtubule structure or causes apoptosis. Additionally, it interacts with lipoic acid to form protein adducts (cysteine, histidine, and lysine target acids), which are present in AD brains [34]. Therefore, neurodegenerative disease and oxidative stress may be causally related.

**Figure 1:**
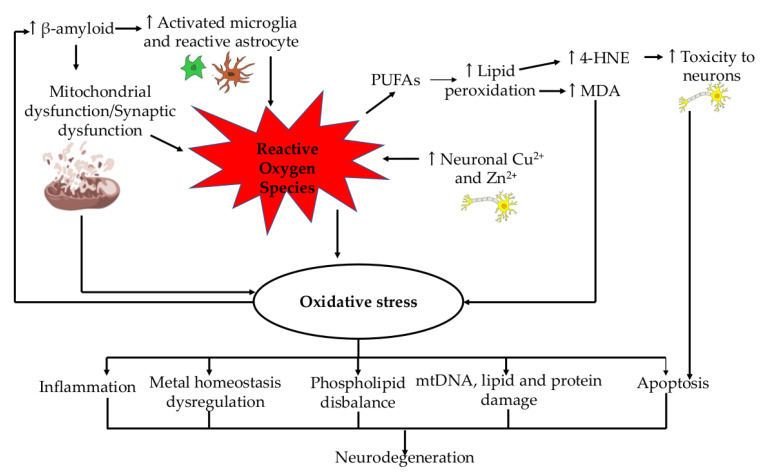
ROS production (red area) as a significant player in the cycle of events in mitochondria (brown area), neurons (yellow/grey area), microaglia (green area) and astrocytes (orange area) leading to neurodegeneration. Abbreviations: ↑, increase; ↓, decrease; PUFAs, poly-unsaturated fatty acids; 4-HNE, 4-Hydroxynonenal; MDA, malondialdehyde; Cu^2+^, copper ion; Zn^2+^, zinc ion; mtDNA, mitochondrial DNA.

Age-related disorders are impacted by the cytoprotective and physiological functions of the antioxidant gas transmitter hydrogen sulfide (H_2_S) [35]. The same precursors as glyoxal and methylglyoxal, as well as N-(carboxymethyl)-lysine (CML) or N-(carboxymethyl)-cysteine (CMC), can also be used to form advanced glycation end products (AGE) and advanced lipoxidation end products (ALE) because of oxidative stress. Glycoxidation processes lead to the formation of AGEs, whereas lipid peroxidation reactions lead to the formation of ALEs. These approaches lead to a very complex amalgamation of interconnected molecules. They include 4-oxo-2-nonal (ONE), HNE, and other highly reactive electrophilic aldehydes and their derivatives [36]. They alter, cross-link, oligomerize, and aggregate due to their interaction with free protein amino groups. In ageing and long-term age-related disorders, these actions lead to intracellular damage, impaired cell function, and ultimately cell death. Nitric oxide synthase is induced by AGEs (iNOS). According to immunochemical tests, AGEs and iNOS were shown to co-occur in astrocytes and microglia [37]. A summary of the role of ROS production as a significant player in neurodegeneration is presented in Figure 1.

## 3. Transcriptomic Alterations in Alzheimer’s Disease

The primary cause of AD pathology, Aβ-derived oligomers, changes the spine’s composition and shape while impairing synaptic plasticity. Soluble Aβ impairs the retrograde BDNF/TrkB signaling. The selective recruitment of a single mRNA into axons and their local translation were established by applying Aβ1-42 to separate axons in tripartite microfluidic chambers. Aβ-1-42-treated AXONS transcriptome study identified mRNAs for several AD-related genes, including transcripts for APP, ApoE, Clu, which controls the generation and metabolism of tau pathology, and FERMT2 [38]. These transcripts’ axonic rise in response to Aβ-1-42 therapy demonstrated that these proteins can affect the amyloid illness downstream. Moreover, after receiving Aβ1-42 treatment, transcript-increased transcriptome coding for transcription factor 4 (ATF4) activation was found. In the subiculum and entorhinal cortex, AD patients do, however, exhibit elevated levels of axonal ATF4 protein and transcripts. The results of hippocampal neurons and the adult mouse brain are closely mirrored in these observations, which is unsurprising given that these brain regions are particularly vulnerable to AD. The results of the study suggested that a neurodegenerative stimulus’s ability to transport and translate axonal mRNA may significantly impact how quickly the pathologic changes connected with AD spread [38].

To categorize the complicated cellular alterations in AD brain disease, 48 people with varying degrees of AD pathology had their prefrontal cortex’s 80,660 single nucleus transcriptomes examined and analyzed [39]. Identifying transcriptional subpopulations in six primary kinds of brain cells led to identifying disease-related subpopulations that are defined by myelination, inflammation, and neuronal survival. Early on and very cell-specific alterations most closely related to AD occurred. On the other hand, the late-stage changes were widespread across all cell types and predominantly contributed to global stress reactions. Unexpectedly, female cells were overrepresented in subpopulations linked with AD, and oligodendrocytes, among other cell types, showed significantly distinct transcriptional responses depending on gender [39].

Accurate spatiotemporal transcriptome control is necessary for the ageing process, and changes in gene expression in the ageing brain have been thoroughly examined. But the mounting body of evidence suggests that ageing changes depend on gene expression and the coordination of several transcriptional controls [40]. Up to 95% of human multi-exon genes are subject to alternative splicing, a crucial process for enhancing the spatiotemporal complexity of the transcriptome that results in many mRNA transcripts from a single gene [41]. The transcriptome is the entire set of transcripts in a single cell or tissue type. Transcriptomic analysis typically aims for the differential identification of genes expressed in different conditions, resulting in a new understanding of the genes or pathways linked to these conditions. It requires an effective statistical tool with a multi-compared test to explain global changes in the expression of thousands of genes [42]. Several techniques, including microarray screening and RNA sequencing techniques, have been developed to analyze the gene expression. Genetic analysis has enabled the extensive use of array technologies, whether for genotyping, linkage analysis, or gene expression [43].

The Affymetrix GeneChip array is frequently used in gene expression analysis, enabling the investigation of gene-level expression. Patel et al. [44] have kept track of the differentially expressed genes (DEGs) in the brain tissues of AD patients. This study compared the transcriptomic patterns of various diseases, such as Huntington’s disease, bipolar disorder, and schizophrenia, using Affymetrix and Illumina arrays. This study identified several DEGs that are particular to AD and may be important for comprehending AD-related mechanisms. The study was based on predefined probes and publicly available transcriptomic data, but the sample size was small, and it might be challenging to map all of the brain’s tissues [44]. Li et al. [45] compared the data from the brain and blood transcriptomes, followed by Affymetryx array platforms, and discovered several pathways that were shared by both, including pathways related to mitochondrial or oxidative stress. However, larger cohorts may facilitate the validation of this study [45]. The human choroid plexus (CP) gene’s expression has been examined using transcriptome-wide Affymetrix microarrays to identify disease-related variations. Four patients with fronto-temporal dementia (FTD), three patients with HD, seven patients with advanced AD (Braak and Braak III–VI) and six healthy controls (Ctrl) had their RNA recovered from post-mortem samples taken from all of the lateral ventricular CP [46]. Comparing the CP of advanced AD patients to controls revealed a significant difference in gene expression. The most frequently found immunological and metabolic pathways in elevated genes included JAK-STAT, mTOR, interferons, cytokines, acute-phase response, and cell adhesion. Individuals with FTD and HD exhibited a variety of changes in gene expression, but there were also apparent differences between the two disease populations. In AD and HD (but not in FTD), several neuroimmune modulatory interferons, such as IFI-TM1, IFN-AR1, IFN-AR2, and IFN-GR2, were markedly elevated [46]. Increased expression of VEGF signaling and immune response proteins, such as interleukins, amplified changes in the expression of genes linked to AD but not in HD and FTD. Patients with HD and FTD had different cadherin-mediated adhesion upregulations [46]. The advantage of array technology is that, compared to sequencing methods, arrays may require less labor overall, and sample preparation and data screening may be much easier. The drawback of arrays is that they need a reference transcript from an expressed sequence tag library or an annotated genome sequence. Only the SNPs, DEGs, splice variants, or miRNAs that the probes were made for can be found using arrays. Additional or novel variations and potential candidates cannot be found using arrays [43].

RNA sequencing (RNASeq) tools are next-generation sequencing (NGS) technologies used to screen transcriptomes for diseases. RNASeq techniques can potentially be used for gene expression profiling, alternative splicing analysis, sequencing targeted RNA molecules, and single-cell sequencing [47]. Although it may be possible to sequence RNA molecules directly, most techniques are based on DNA sequencing. Various RNA sequencing tools may be available, including Roche 454, Illumina, Helicos (based on DNA polymerase), and PacBio, SoLid (based on ligase). The experiment’s goal may influence the choice of platforms. While Illumina or SoLid require multiple copies (ensemble-based) of DNA or RNA, Helicos or PacBio may be useful for single-molecule detection. A higher error rate risk may exist when comparing single-molecule sequencing platforms to ensemble-based approaches. In the case of miRNA sequencing, the low error rate may be crucial. Illumina and Solid might offer a deeper level of sequencing. PacBio or Roche454 may be better if longer reads are required [48]. Annese et al. [49] identified the platform- and gene-regulation in late-onset AD (LOAD) patients by examining brain DEGs and miRNAs using Illumina platforms. More than 2000 DEGs involved in various processes, including vesicle trafficking and neural system regulation, were found in this study. Among LOAD patients, they also discovered novel miRNA clusters. This investigation might help us comprehend the LOAD pathways better. This study’s limitation was its inability to offer information on the earliest stages of neurodegeneration [49]. The DEGs between hippocampal mRNAs from AD patients and controls by Illumina HiSeq2000 at 2 × 50 bp were the focus of a study by van Rooij et al. [50]. They used gene-annotated modules to perform integrative network analysis, but this research may also need to be validated [50]. Postmortem brain samples with both high amyloid deposition and low or no amyloid pathology were examined by Mathys et al. [39]. The single-nucleus RNA sequencing (snRNA-seq) used in this study was droplet-based. A more precise picture of the expression changes associated with AD and the complexity of gene interactions can be obtained from single-cell RNA expression analysis. However, since it may be difficult to distinguish between protective and pathogenic pathways, additional research on single-cell RNA sequencing (scRNA-seq) may be required [39]. The advantages of RNASeq techniques include their typically straightforward workflow. Sequencing may be performed on various platforms, and bioinformatics techniques may make analysis simple [43]. The main drawbacks of array technologies were intended to be overcome by RNASeq techniques. Hybridization in arrays can be challenging; cross-hybridization can happen when sequences are similar, producing false-positive results; a priori knowledge of the sequences is required; and, finally, the qualification of lowly and highly expressed genes can be challenging. RNASeq could offer higher throughput, the more accurate quantification of gene expression, and the discovery of novel DEGs, non-coding RNAs, or alternatively spliced genes [43]. RNASeq techniques may face several difficulties, including problems with repetitive sequence elements or alternatively spliced genes. Dealing with hardware and bioinformatics tools and processing data can be difficult [43]. Most brain transcriptome methods have limitations in that they do not emphasize cell-to-cell variability or reveal the heterogenic expression pattern among cells from various tissues. By profiling the gene expression in different individual cells, single-cell transcriptome sequencing may be used to identify the transcriptional diversity of the human brain [51]. ScRNA-seq may offer a greater resolution of the expression pattern at the single-cell level, but the data may be noisier and more complex. Additional bioinformatics tools are required to analyze and verify the accuracy of the scRNA-seq data [51]. A summary of the relevant results from micro-array and scRNA-seq has been included in Table 1.

Despite the relatively high accuracy of RNASeq and microarray analyses, some publishers may insist on data validation. When data sets from the RNASeq and Affymetrix platforms were compared using the same set of samples, it was discovered that there were substantial correlations between the gene expression profiles generated by the two platforms [52]. Compared to arrays, sample preparation may be more difficult with RNASeq technologies [48]. However, it has also been shown that RNASeq is more effective at finding low-abundance transcripts, spotting genetic variations, and differentiating physio-logically significant isoforms. RNASeq has also demonstrated a higher dynamic range than a microarray, enabling the identification of genes with higher variability and more differential expression [52]. ScRNA-seq is a useful technique for analyzing the effects of pharmaceutical therapy on individual cells in complex tissues. This application calls for high precision and quantitative accuracy beyond what is currently practical for solely qualitative single-cell analysis [53].

## 4. Vitamin E

It has been established that vitamin E, especially α-tocopherol (αTP), is necessary for preserving the integrity of the CNS. The brain is particularly vulnerable to oxidative stress because it requires more oxygen due to its high metabolic demand. The danger of lipid peroxidation in the brain is also increased by the abundance of polyunsaturated fatty acids (PUFAs) [54]. Four tocopherols and four tocotrienol isomers, each having an isoform of α, β, γ and δ, make up vitamin E. All eight isomers can be found in various foods, including cereals, nuts, seeds, and vegetable oils. The predominant isoform of vitamin E typically found in supplements is α-tocopherol. Adults should consume no more than 1000 mg/day of vitamin E as their recommended daily maximum, and only 15 mg/day of additional vitamin E should be consumed by adults [55]. A phenolic hydroxyl group on the chromanol ring, which is central to vitamin E’s antioxidant activity, can donate an atom of hydrogen and thus displace a variety of free radicals, including those that produce ROS and vitamin E radicals. The free radicals produced by vitamin E can interact with other free radicals found in lipids or can be neutralized by vitamin C. Through this mechanism, vitamin E neutralizes peroxyl radicals and guards against lipid peroxidation, especially polyunsaturated fatty acids, which are crucial for the defense of cell membranes [56].

Vitamin E is regulated by several genes, including xanthine oxidase, collagenase, SRA and CD36 scavenger receptors, tropomyosin, α-TTP, and CYP3A. Vitamin E deficiency alters many genes in mice and rats. When vitamin E is absent, the body should produce substitute antioxidants to fill the gap. No antioxidant gene is overexpressed as a result. In mice, vitamin E is the only factor that controls several T-cell genes. It is important to note that the regulation of gene expression seems to be tissue-specific [57]. These tissue-specific genes controlled by vitamin E create proteins involved in the elimination of β-amyloid and advanced glycated end products, nerve growth, hormone metabolism, apoptosis, and dopaminergic neurotransmission [58]. Fruit, vegetables, nuts, and phytochemicals are thought to change the neuro-generational processes connected to the formation of misfolded proteins and neuro-inflammation, preventing disorders like AD and PD. The common etiology of hazardous misfolded proteins in neurodegenerative disorders suggests that many of these diseases could benefit from a complementary therapeutic strategy that prevents the disposition of such proteins [59].

## 5. Effect of Vitamin E’s Transcriptomic Alterations in Alzheimer’s Disease

Age-related oxidative stress can contribute to the development and progression of AD. It has been demonstrated that vitamin E has anti-inflammatory and antioxidant properties that may protect against neurodegeneration, prevent the onset or advancement of this illness and alleviate the cognitive loss that occurs naturally as we age [60,61,62]. Aβ (1–40) hippocampal injection impaired memory for novel object recognition in rats and increased oxidative stress, but vitamin E supplementation for 14 days helped reduce oxidative stress by lowering malondialdehyde (MDA) and raising superoxide dismutase (SOD), which was linked to the loss of fewer neurons and better memory [63].

Efforts to develop a vitamin-E-based treatment for Alzheimer’s disease have continued. Many studies are still being conducted to determine the effectiveness of various vitamin E forms in preventing AD progression. In one study, the effects of p38 signaling on AD and the protection offered by the vitamin E analogue Trolox were also investigated. In addition to its antioxidant capabilities, vitamin E has been found to have a variety of qualities that can modify cell signaling pathways [64]. Vitamin E treatment was also demonstrated to significantly raise the mRNA levels of the scavenger receptor CD36 and reduce the elevated expression of CD36 mRNA in the aorta of rabbits fed cholesterol [65], which facilitates the elevated expression of β-amyloid toxicity via lipid peroxidation products [66]. The findings of a study by Pahrudin Arrozi et al. [67] also implied that alpha-tocopherol (ATF) and gamma-tocopherol (GTF) might shield the cells from β-amyloid toxicity by energizing and repressing the gene expression involved in the control of the APP non-amyloidogenic and amyloidogenic processing pathway, respectively. This defense mechanism could protect the cells against β-amyloid toxicity. In addition, daily tocotrienol-rich fraction (TRF) supplementation given to APPswe/PS1dE9 double transgenic mice for 10 months reduced β-amyloid immunoreactive depositions and thioflavin-S-positive fibrillar-type plaques in the brain and eventually enhanced cognitive function in the novel object recognition test compared to control APPswe/PS1dE9 mice. Similarly, TRF attenuated the affected biological processes and pathways and successfully stopped the AD conditions by modulating several genes in the hippocampus of APPswe/PS1dE9 double transgenic mice [68].

**Figure 2:**
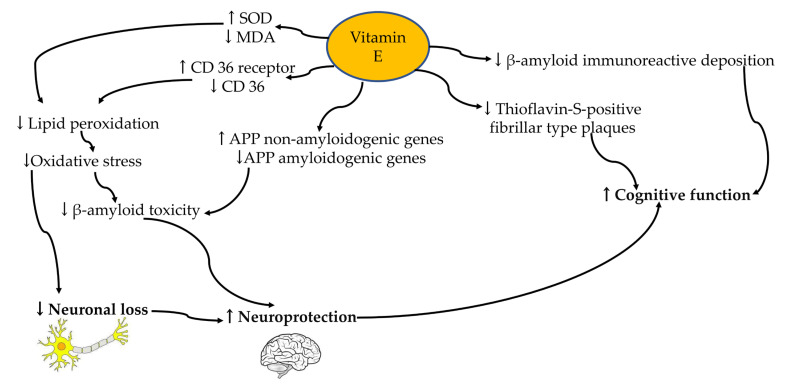
Vitamin E (yellow circle) regulation on neuroprotection mechanism and cognitive function in the neurons (yellow/grey area) and brain (black/white area). Abbreviations: ↑, increase; ↓, increase; MDA, malondialdehyde; SOD, superoxide dismutase; APP, amyloid-beta precursor protein.

In a double-blind, placebo-controlled, parallel-group, randomized clinical trial (The TEAM-AD VA Cooperative Randomized Trial) by Dysken et al. [69], it was found that 2000 IU/d of alpha-tocopherol supplementation reduced functional decline in patients with mild to moderate Alzheimer’s disease. However, Kryscio et al. [70] discovered that a low dose of vitamin E (400 IU/d) supplementation in 7540 asymptomatic older men did not prevent dementia. This demonstrates that vitamin E treatment can sometimes improve cognition but not always. The replacement of lost neuronal networks, the very different nutritional status of the patients at baseline, the varying lengths of time for brain compensation in each individual, and the antioxidant effect of vitamin E in each individual, among other factors, could all contribute to treatment failure [56]. A summary of vitamin E regulation has been included in Figure 2.

## 6. Conclusions

Deep sequencing-technology-based transcriptome profiling offers insight into frequently changing pathways regardless of the underlying genetic or epigenetic factors. As a result, it is thought to be an excellent technique for studying the molecular mechanisms underlying AD pathogenesis. Technologies developed by transcriptomics, such as arrays and sequencing, have the potential to provide precise data on gene DEGs, alternative splicing, or miRNAs. However, maintaining the quality of RNA samples may be difficult, and transcriptome profiles from different tissues (such as blood vs. brain) may differ. Furthermore, blood mRNA may be unstable, and obtaining samples of human brain tissue may be difficult because brain samples are typically obtained post-mortem or through brain biopsy.

The number of people affected by AD is steadily increasing; therefore, developing a treatment capable of preventing or delaying AD progression is necessary. Evidence suggests that oxidative stress plays a significant role in AD pathology. The current literature demonstrates that ß-amyloid contributes to neurodegeneration in AD by increasing ROS, leading to lipid peroxidation and increased oxidative stress. Increased oxidative stress leads to a further increase in ß-amyloid. Given this, antioxidant compounds may be helpful in the prevention/treatment of AD. The preclinical evidence reviewed suggests that vitamin E supplementation may benefit AD because it can counteract ß-amyloid toxicity and oxidative stress, resulting in lower neuronal loss, increased neuroprotection, and improved cognitive function. The clinical trials, on the other hand, produced contradictory results regarding vitamin E’s efficacy in AD. The reviewed studies suggested that high vitamin E doses seem to have better results. However, more research is needed to determine the effectiveness of vitamin E in AD. Trials comparing various vitamin E dosages and forms may be beneficial.

## Figures and Tables

**Table 1:** Summary of gene expression studies using microarray and scRNA-seq in AD.

Author	Study Design	Brain Region	Result
Patel et al. [44]	Study type: Meta-analysis with 1501 (746 AD, 755 controls) male and female subjects using microarray (Affymetrix and Illumina) and scRNA-seq technology. Number of patients: 1501 (746 AD, 755 controls) Sex: Male and Female Technology: microarray (Affymetrix and Illumina); scRNA-seq	Frontal Lobe Temporal lobe Parietal lobe Cerebellum	- Identified 323, 435, 1023, and 828 DEGs specific to the AD temporal lobe, frontal lobe, parietal lobe, and cerebellum brain regions, respectively. - Seven of these genes consistently altered across all AD brain regions with the SPCS1 gene expression pattern replicating in RNA-Seq data. - Nineteen genes altered specifically in AD brain regions affected by both plaques and tangles, suggesting possible involvement in AD neuropathology. - Biological pathways involved in the metabolism of proteins and viral components significantly enriched across AD brains.
Li et al. [45]	Systematic analysis with 245 AD, 143 MCI and 182 control male and female subjects using microarray (Illumina) technology.	Occipital Visual Cortex Dorsolateral Prefrontal Cortex Superior Temporal Gyrus Anterior Cingulate Inferior Temporal Gyrus Superior Parietal Lobule Putamen Posterior Cingulate Cortex Nucleus Accumbens Inferior Frontal Gyrus Caudate Nucleus Precentral Gyrus Amygdala Para hippocampal Gyrus Temporal Pole Middle Temporal Gyrus Frontal Pole Hippocampus	- In 998 DEGs in the brain of AD and MCI subjects, over 77% had the same regulation directions across tissues and disease status, including the known *ABCA7*, *TYK2* and *TCIRG1.* - Machine learning classification model containing *NDUFA1*, *MRPL51*, and *RPL36AL* implicating mitochondrial and ribosomal function discovered, distinct between AD patients and controls with 85.9% of the area under the curve and 78.1% accuracy. - Findings suggest mitochondrial dysfunction, NF-κB signaling and iNOS signaling are important dysregulated pathways in AD pathogenesis.
Stopa et al. [46]	Comparative study with 6 Ctrl, 7 AD, 4 FTD and 3 HD male and Female subjects using microarray (Affymetrix) technology.	Choroid plexus	- ↑ IFI-TM1, IFN-AR1, IFN-AR2, and IFN-GR2 in AD and HD - ↑ VEGF and interleukins in AD
Mathys et al. [39]	Longitudinal cohort study with 48 AD (24 elevated Aβ and 24 individuals with no or very low Aβ) male and female subjects using scRNA-seq technology	Prefrontal cortex	- Transcriptionally distinct subpopulations across six major brain cell types identified. - Strongest AD-associated changes appeared early in pathological progression and highly cell-type-specific genes. - Genes upregulated in the late stage common across cell types and primarily involved in global stress response. - Overrepresentation of female cells in AD-associated subpopulations, - Substantially different transcriptional responses between sexes in multiple cell types, including oligodendrocytes.
Annese et al. [49]	Cohort study with 18 (6 controls, 6 AD and 6 PD) males subjects using scRNA-seq (Illumina HiSeq2000) technology	Hippocampus Temporal gyrus Frontal gyrus	- miR-132/212 cluster deregulated in each brain region in LOAD patients, consistent with miRNAs playing a role in AD pathogenesis. - miR-184 can target the 3′UTR *NR4A2* (known to be involved in cognitive functions and long-term memory; expression levels are inversely correlated with miR-184 in the hippocampus). - ↓ RNA editing in LOAD hippocampus, 14 recoding sites significantly and differentially edited in 11 genes.
Van Rooij et al. [50]	Cohort study with 18 (10 controls and 8 AD) male and female subjects using scRNA-seq technology	Hippocampus	- In total, 735 DEGs clustered into 33 modules - In total, 27 modules enriched for signal transduction, transport, response to stimulus, and several organic and cellular metabolic pathways. - Ten modules interacted with previously described AD genes.

Abbreviations: ↑, increase; ↓, increase; AD, Alzheimer’s Disease; LOAD, Late-Onset AD; DEGs, differentially expressed genes; FTD frontotemporal dementia; HD, Huntington’s disease; MCI, mild cognitive impairment; IFI-TM1 Interferon-induced transmembrane protein 1; IFN-AR1, interferon alpha receptor subunit 1; IFN-AR2, interferon alpha receptor subunit 2; IFN-GR2, interferon-gamma receptor 2; scRNA-seq, single-cell RNA sequencing; VEGF, vascular endothelial growth factor; NF-κB; nuclear factor kappa B; iNOS, inducible nitric oxide synthase.

## Data Availability

No new data were created or analyzed in this study. Data sharing is not applicable to this article.
